# A Text-Book of Physiological Chemistry

**Published:** 1902-04

**Authors:** 


A TEXT-BOOK OF PHYSIOLOGICAL CHEMISTRY. For Students of Medicine and Physicians. By CHARLES E. SIMON, M. D., of Baltimore, Author of "Simon's Clinical Diagnosis," etc. Octavo volume of 452 pages. Philadelphia and New York: Lea Brothers & Co. 1901. [Price, cloth, $3.25 net.]

The author of this work needs no introduction to the American profession, as his excellent work on Clinical Diagnosis has been before the public for several years and has passed through numerous editions. His success at book-making enticed the author into a neighboring field and another excellent work is the result of his careful labors and researches.

Dr. Simon is an admirably clear writer and teacher, and has adapted his book not only to the needs of students, but also to those of the practitioners who may have been unable to devote to the subject the study which it merits. The volume will also serve as a guide in collegiate or private laboratories.

The first section of the book gives a general survey of the origin and chemical nature of the three great classes of foods and of the products of their decomposition. In the second section, are treated the processes of digestion, resorption and excretion. The third is devoted to the chemical study of the tissues and various organs of the body, the products of their action and their relation to physiological function.

The author discusses the elementary composition of the albumins, fats and carbohydrates, gives the different characteristic reactions, their solubility, behavior to acids and alkalies, and the chemical composition of their compounds and derivatives. Under the second head are discussed the juices of the body, the process of digestion, either of the carbohydrates, albumins or fats. The chapter devoted to the urine is unusually complete, the newer tests are carefully described, and their importance pointed out.

The chemistry of the different organs of the body occupies the concluding chapters, and is perhaps the most interesting part of a most interesting book. To many the advances in therapeutics must be in line with the chemistry of the different organs and progress will be most satisfactory. Dr. Simon deserves the thanks of the profession for his painstaking labor and should be accorded its cordial support. W.C.K.

